# Selections and Abstracts

**Published:** 1900-05

**Authors:** 


### *SELECTIONS AND ABSTRACTS.*

---

ANKYLOSTOMIASIS THE CAUSE OF PUERTO RICAN FEVER, OR TROPICAL OR EGYPTIAN CHLOROSIS, OR SEVERE ANEMIA, OR CACHEXIE AQUEUSE.

By permission of the Surgeon-General United States Army, the *New York Medical Journal,* April 14, 1900, publishes the Report of Assistant Surgeon Bailey K. Ashford on "*Ankylostomiasis in Puerto Rico,*" which contains suggestions that will undoubtedly prove of inestimable value. He says:

One of the first observations made by professional men here is the prevalence of anemia, especially among the poor. This is at first attributed usually to starvation or poor food, then to malaria, and then to the "climate." The ignorant peon treated himself by purging, with beneficial effects for a time and a relapse to previous conditions soon afterward. Iron and arsenic have been prescribed largely, but with little benefit. Some physicians frankly declare it beyond their power to cope with the disease, which they regard as a pernicious, progressive anemia of obscure origin. Stools have been examined, but, no worms being evident, this, as a cause, was dismissed. I was led to examine the feces for the ova of *Ankylostoma duodenale*, and found them in great numbers. Soon after, a large dose of thymol brought away the parasites, male and female. No sooner had I stated my results to the physicians of this city than they agreed as to diagnosis, and verified the parasite and its eggs. Their testimony is as follows:

1. This disease is the most destructive and general disease of Puerto Rico.

2. It is found typically and very frequently among the poor and badly fed.

3. Most cases are similar.

4. Bad food and bad hygiene are responsible for much of its power for evil.

5. Blood foods have never exercised more than a temporary influence on the course of this disease.

6. Improvement follows purgation.

7. Up to this time the existence of this parasite had not been proved on this island, or, if proved, not within their knowledge.

In studying this disease twenty cases typical of "Puerto Rican anemia," or "tropical chlorosis," were selected from the provisional field hospital for indigent and sick Puerto Ricians, established after the flood of August 10, 1899, in this city.

1. *Family History.*—Most patients give a history of deaths in the family from a like disease. At times this history is truly appalling. Many claim the deaths to have been due to "malaria" or "diarrhea" or "obscure fever." It is fair to suppose, inasmuch as the disease is often marked by irregular fever, with intermissions, that their diagnosis may be questioned. But questions as to chills are extremely unsatisfactory. There is much malaria here in the lowlands. I have followed such cases through their course, but the testimony of local physicians coincides with mine, that malarial organisms in the blood are not so often seen as would be supposed. Chills, then, are not so frequent; there are few

"ague cakes"; the pallor is not that of malaria, and the scleræ are not icteric. The most suggestive fact outside of blood examination is that the cases come from the mountains and the valleys; some of the very worst cases I have seen came from highly salubrious mountain districts. Nevertheless, I hesitate to affirm that many cases of malarial cachexias do not exist to swell the sum of anemics here.

2. *Previous History of Patient.*—The diet is a powerful factor in turning the scale against the unhappy victim of ankylostomiasis. Rightly the physicians here quote its influence. Personally, I have eaten and slept in all parts of this island, not alone on the frequented roads, but in those rarely visited by strangers, and can submit my testimony to that quoted in support of this influence. The relation of the daily life among the working classes has been confirmed in talking with many owners of sugar and coffee plantations and their employees. They rise at from 4 to 6 A.M. Some take a little black coffee, some boiled water and sugar, some nothing. They work till eleven, when they breakfast on about four ounces of codfish and a few pieces of plantain. They return to work at 1 and continue till 5 P.M. Dinner is composed of rice and beans; some have only boiled rice with lard, and some boiled rice alone. They get plenty of bad rum and some bad wine. This seems a slight enough diet, but the hurricane deprive them of even this, and the sick poor came drifting down on Ponce. Bathing is not often practiced. Feces are distributed over the earth whereever the individual happens to be while at work, or in a little shack when at home, but directly on the ground always. Indeed, feces pollute their very houses. Ponce is a town of, perhaps, forty thousand inhabitants, yet it has no sewerage, and is in the lowlands near the sea. Closets and kitchens are in conjunction in many houses. The water soon takes up its quota of whatever is noxious. Those who are clean in their habits—and the educated classes are a most cleanly people—are polluted by the filth of the poor and ignorant. The configuration of this island is one of steep mountains and deep ravines, with broad plains near the sea. Heavy rains wash the larvæ from each fecal deposit into these watercourses, and this muddy water is probably one source of contamination. Contaminated earth on the hands of laborers is another; fouled garden is another. The larvæ have not yet been demonstrated in the water or mud. The drinking-water of nearly all well-to-do people is filtered, and in this class we do not find so great a preponderance of this disease.

3. *Subjective Symptoms.*—It is difficult to obtain a history of the disease from its inception, for many have it from infancy. Generally it is possible to obtain some such history as this: A variable appetite, some nausea and vomiting, pain in epigastrium, either constipation or diarrhea (or these may alternate), sometimes dysentery; swellings of the feet and ankles, no loss of weight, sleeplessness, restlessness, tinnitus aurium, giddiness, faintness, severe headache, palpitation of the heart, progressive debility; little perspiration, but kidneys active; fever sometimes, but no chills. I have not been able to get a history of geophagism or of intestinal hemorrhage described by some authors. Sometimes the patients improve for a time after medication, but not permanently.

4. *Objective Symptoms.—Pallor:* The conjunctivæ, lips, tongue, gums, nails, and cheeks are in some cases perfectly pallid, the mucous membranes especially being of a deathly white. The skin is generally a pasty yellow, a dirty brownish-gray, or a grayish-white. *Expression:* A passive expression is often seen, and its peculiar character is heightened by puffiness of the eyes and bloating of the face. *Edema:* This is simply the usual accompaniment of severe anemia. Practically, every variety is seen, the chief being, in order of importance, odema of the feet and ankles, odema of the face, ascites, and odema of the scrotum. *Hypostatic congestion of the lungs* exists often. The important point is that with this disease there may be *emaciation.* This has not been present often in my cases; on the contrary, the patients are apparently well nourished. *Anemic ulcers* are sometimes seen on the legs, and an incorrect accusation of syphilis may be made. Corneal ulcers are at times seem. One of my cases presented corneal ulcers of both eyes. *Respiratory symptoms:* Generally none from this disease save in increased rapidity of breathing from anemia, serous accumulations, or hypostatic congestion. *Liver:* No constant symptom. *Spleen:* No constant symptom. *Heart:* These symptoms are very aggravated; signs of a pernicious secondary anemia. *Pulsating vessels:* Both jugulars, superficial veins of the arm, and vessels about the root of the neck and heart in severe cases, with greatly dilated heart; pulsating suprasternal and supraclavicular regions and diffused pulsations in the anterior thoracic wall. All kinds of deductions might be made by a careless observer. *Hemic murmurs* are almost constantly present, and are in many cases heard in the veins of the neck.

*The urine:* No albumin is found and the specific gravity is constantly low. The pulse is weak, rapid, soft, and compressible.

*The blood:* The following deductions are drawn from blood examinations:—

1. A severe anemia, falling as low as that of Addison's anemia in count of red cells in some cases.

2. A very low hemoglobin average and a very low color index.

3. A marked eosinophilia in some cases. Forty per cent. reached in one case. This follows the observation of Neusser.

4. No leucocytosis common to the disease itself. Leucocytosis recorded is always apparently due to complications.

5. Frequent presence of normoblasts, and in some cases megaloblasts, but never a majority of megaloblasts.

6. Poikilocytosis common. Manson denies this.

7. Utter unreliability of blood foods without removal of the cause, the ankylostoma.

This blood examination was the first line of research taken up, and as soon as anemia was proved, the patient was given blood tonics, with temporary supporting treatment suited to the indvidual case, with the idea that the patient might be carried along until the true cause could be discovered. Of course, now, all treatment has been substituted by anthelminthics, chiefly male fern and thymol, and the blood and heart tonics will be again tried when the eggs have disappeared from the feces. I was led to examine the stools carefully from the high eosinophile count, and it is certainly evident that trichinosis has a rival for high counts in ankylostomiasis.

The ankylostoma was found in all cases save one, a case of tuberculosis pure and simple. This patient was chosen to represent a contrast, and I think he does. There is true leucocytosis, and the eosinophiles are not much in evidence. Moreover, the red cell count is much higher than all the others, as is the hemoglobin record.

The histories of this disease have been made up to show what percentage of the people have the disease in certain countries: Twenty-five per cent. in Egypt, twenty per cent. in Maitland, fifty-two in Madras, thirteen per cent. in Kioto, Japan; but no percentage can be cited as yet for this island. Dr. B. Scheube of Greiz speaks in his work of its existing in the Antilles, but no island is specified, nor is the extent of the disease stated. From my own observation and from the opinions of the resident physicians of the island, I believe it to be wide-spread and destructive. Only twenty cases have been examined, yet all, save one, have given me the ova of this parasite in large numbers. As the twenty cases were chosen at random from hundreds more just like them clinically, and as the one exception noted was chosen only for contrast, I am convinced that further investigation will show that the disease has killed its hundreds, and that it is curable and preventable. The proof of its prevalence lies naturally in the hands of all scientific physicians of this island. I cannot further judge than from a short experience and the positive evidence of nineteen cases submitted.

A glance at its geographical distribution is appalling. Egypt is so full of it that it is known as Egyptian chlorosis, and forms the great basis for rejection of recruits for the army. The French, of the French Antilles, call it *cachexie aqueuse*, and recognize its full importance; and literature is full of its ravages in South America. Thornhill regards it as of greater importance in Ceylon than cholera.

There is in Puerto Rico a dense population in a small country. In a space of about a hundred miles by sixty we have probably over a million people. Of the working class it cannot be denied that a large percentage have anemia; and, should the future verify my suspicion, means are at hand to increase not only the well-being of those now suffering, but to insure to the owners of large haciendas of coffee and sugar a better class of labor; to insure to the army protection from the invaliding from anemia of such troops as are enlisted here; to insure protection against the disease to our American troops; to relieve the State and the hospital here from the expense of caring for a large number of anemics who are now slowly dragging on to a fatal end. Perhaps our own sick reports will unfold some additional facts.

I mention here only such possibilities as have occurred to my mind; but it is a significant fact that, though it is present in Germany, Scheube notes that it is confined to a few cases. In other words, it appears to assume only such proportions as a country will allow it to assume.

I have given thymol in several cases with the always easily demonstrated presence of the parasite. From the exceeding kindness and the scientific spirit shown by the local doctors, I cannot doubt that it will be but a short time before measures will be taken, if there is sufficient extent of the disease found, to alleviate the conditions.— *Virginia Medical Semi-Monthly.*

---

### THE UTERINE DOUCHE.

Edward P. Davis, A.M., M.D., of Jefferson Medical College, is the author of a paper upon the indications for and method of employing the douche, which appears in the March number of the *American Gynecological and Obstetrical Journal.* After a discussion of the dangers from sepsis, and other undesirable results of careless douching, the author proceeds as follows:

"The indications for an intra-uterine douche are precisely those conditions which are at the same time its dangers, namely, septic infection and hemorrhage.

"In the former it seems rational to remove from the uterus retained and decomposing blood-clot, placental tissue, membranes or purulent secretion and bacteria. In cases which proceed normally, the uterus drains itself after labor by its intermittent contractions, and its cavity, being aseptic, the lochial discharge is removed with sufficient thoroughness. When, however, infection is present, the contractions of the womb are less active, its walls are relaxed, the lochial discharge is retained and partially absorbed, and the patient undergoes a gradual auto-intoxication. When the womb is infected, as shown by an abnormal lochial discharge or a lack of the normal discharge, with fever and rapid pulse, the indication is clear to thoroughly cleanse the interior of the womb.

"This may be done by the use of a stream of sterile fluid, or by the use of dilute antiseptic solutions. In all cases in which the patient is profoundly depressed and has suffered from loss of blood, it is well to employ normal salt solution rather than one of the carbolic derivatives. Mercurial solutions should not be used within the uterus. For the first cleansing of the uterus a thorough exploration of its cavity is necessary, as well as a douche. The finger may be employed for this purpose, although it will rarely be sufficiently long to accomplish the result thoroughly. In our experience it is much better to employ a douche-curette, thoroughly but gently exploring the uterine cavity and douching it at the same time. This procedure rarely requires the use of an anesthetic. The external parts and the vagina must be made aseptic before the uterine cavity is entered, and the patient may be conveniently placed across her bed, her hips projecting slightly over its edge. By attaching a fountain syringe to the curette a copious douche may be given. From one to two gallons of fluid may be used to advantage. In septic cases we prefer not to employ a sharp-edged instrument, nor to use forcible curetting, as we wish to interfere as little as possible with the zone of resistance in the uterine tissue and with the thrombi closing its sinuses.

"In septic cases, after the wound has been curetted as described, the physician may desire to give intra-uterine douching a further trial and then he may employ a douche-tube. For our use, we have had made a tube of especially strong glass, having a groove along the distal portion of its under surface. There is no aperture in the tip of the tube, and it possesses but a single curve, along which are holes, which permit the passage of the fluid. The groove serves sufficiently as a channel for the return flow. This tube may readily be boiled, and has given uniform satisfaction. For sterilizing it and also the curettes employed, we use pans of simple construction and convenient size.

Intra-uterine douching has long been considered an efficient means of treatment in post-partum hemorrhage. In this emergency it is not always easy to maintain aseptic precautions. It is better to be prepared for hemorrhage before it occurs than to run the risk of infecting the uterine cavity through a lack of preparation. It is our practice in all cases of labor to sterilize the glass douche tube and to have ready a sufficient supply of sterile or antiseptic fluid to enable us to thoroughly douche the uterus should bleeding occur during labor or soon after its completion. The question naturally arises, by douching the uterus, which is relaxed after the delivery of the child, shall we not remove clots which have formed as plugs to close the sinuses of the uterus? The old teaching was to turn out the large clot, and this we believe to have been correct. A stream of hot fluid will not remove the clots within the sinuses of the uterus, while a considerable mass of coagulum must be removed if the uterus is to contract properly. Intra-uterine douching for hemorrhage is most successful when followed by an intra-uterine tampon of iodoform or sterile gauze. The two in combination empty the womb and make it impossible for a large clot again to form, while stimulating the uterus to contraction.

If a suspicion arises that a portion of placenta or membranes has been retained within the uterus, and that hemorrhage results from this cause, the douche-curette should be employed. An exuberant endometrium and decidua may give rise to hemorrhage, which is best controlled by curetting and douching. The disturbance which follows this slight operation is more than compensated for by the stimulating effects of the hot intra-uterine fluid and by the pressure which the gauze makes if it is found necessary to apply it.

Cases are occasionally seen where sepsis and hemorrhage are combined. In these patients the infection has proceeded to such an extent that the blood is profoundly altered, and its coagulating power greatly reduced. Cases of incomplete abortion, complicated by septic infection, are sometimes among this class. In these patients, prolonged douching and intra-uterine manipulation must be avoided. The use of an anesthetic is dangerous. While it is proper to douche and cleanse the uterus, this should be done as rapidly and gently as possible, the cavity firmly packed with gauze, and the patient stimulated in the most active manner. Such cases are occasionally lost through failure to rapidly perform the necessary manipulations.

"When labor must be terminated by the introduction of the hand or of an instrument within the uterine cavity, and when the patient is to some extent exhausted, so that the contractile force of the uterus is lessened, we believe it to be the safest and best to expedite the removal of the placenta by expression, to thoroughly douche the uterine cavity with sterile water, normal salt solution, or one per cent. lysol at 110 degrees F., and should a tendency to relaxation of the uterus be feared, to conclude the douching by an intra-uterine tamponade of iodoform gauze. When this is removed, the uterus should again be thoroughly flushed with one of the liquids named. No further intra-uterine or vaginal douches in our experience have been required after this treatment.

"In hemorrhage, we have seen excellent results follow an intra-uterine douche given as already described. In septic infection we have seen the best results from thoroughly curetting and douching the uterus so soon as the patient has foul lochia and evidences of uterine infection. In a few cases, a second uterine douche has seemed advantageous. We do not believe it wise to give repeated intra-uterine douches.

"At each case of confinement there should be used a simple sterilizer in which the instruments of the obstetrician may be boiled. A suitable tube for douching the uterus should be in readiness at each case. Its use, however, should be very carefully limited, in our judgment, to the indications given."—*Med. Review of Reviews.*

---

### INTERMITTENT PULSE IN LIFE INSURANCE EXAMINATION.

Recently a young man who had waited in the office a half hour for examination was found with palpitation, and a pulse of ninety, irregular and intermittent. Assuming that his waiting had in a measure quieted the action of the heart, which was likely to reject, and thinking that a little exercise would bring out more clearly the real condition, he was quietly asked to do an errand a few blocks distant while a patient received attention. On his return the writer was surprised to find no palpitation and a pulse of eighty-two, regular and free from intermission. However, on auscultation mitral insufficiency was detected.

Eleven years ago the writer examined a man of thirty-five, whose pulse, on different sittings, skipped a beat at frequent but irregular intervals. He was reported "a good risk, free from organic heart disease, notwithstanding that his nervous and digestive systems are somewhat impaired." He was rejected at the home office, and for a number of years spent much time examining his own pulse, and in consulting traveling doctors. Nevertheless, he still lives, and has continued his work in the wood department of a furniture works, and is to-day in better health than when refused insurance. What disposition shall the examiner make of similar and allied cases? Have not many of them been unnecessarily rejected?

Levan, A Treatise on Medical Examinations for Life Insurance, says "That alteration in the rhythm of the heart, which produces a distinct intermission in its action, whilst frequently found even in healthy persons, suggests, nevertheless, a diseased condition of the valves or orifices, although it may often be difficult to explain the precise cause."

The majority of companies instruct their examiners that an irregular or intermittent pulse should reject. One of the strong companies of to-day in their "Instructions to Medical Examiners," issued more than twelve years ago, says, "An intermittent pulse is not of itself sufficient cause for rejection, as it is sometimes found in the healthy. But an irregular or remittent pulse must ever be taken with suspicion of some grave disorder, either in the heart and great blood-vessels, or in the economy at large. It is sufficient alone to justify rejection."

Osler, Practice of Medicine, third edition, under the heading Arrhythmia, says, "An intermission occurs when one or more beats of the heart are dropped. Irregularity is the condition when the beats are unequal in volume and force, or follow each other at unequal distances." . . . "The causes of these various disturbances of rhythm are thus classified by G. Baumgarten:

(1) Those due to central (cerebral) causes, either organic disease, as in hemorrhage or concussion; more commonly psychical influences.

(2) Reflex influences, such as produce the cardiac irregularity in dyspepsia and diseases of the liver, lungs and kidneys.

(3) Toxic influences; tobacco, coffee and tea are common causes of arrhythmia. Various drugs, such as digitalis, belladonna and aconite, may also induce it.

(4) Changes in the heart itself. (a) In the cardiac ganglia. Fatty, pigmentary and sclerotic changes have been described in cases of this sort, and may have an important influence in producing disturbances in the rhythm; but as yet we do not know their exact significance. They may be present in cases which have not presented arrhythmia. (b) Mural changes are common in conditions of this kind. Simple dilatation, fatty degeneration and sclerosis are most commonly present, the two latter usually associated with sclerosis of the coronary arteries.

The significance of arrhythmia is not always easy to determine. Simple irregular action of the heart may persist for years. The late Chancellor Ferrier, of McGill University, a man of unusual bodily and mental vigor, who died at the age of eighty-seven, had an extremely irregular pulse for almost fifty years of his life. One or two other instances have come under my notice of persons in good health, without arterial or cardiac disease, in whom the heart's action was persistently irregular."

Pepper states that "irregularity of the pulse may be noted for years without any disturbance of the patient's health. In other cases this symptom is of ominous significance. The prognosis is to be based upon the cause of the cardiac arrhythmia. On the whole, the outlook is more grave in any form of myocardial or valvular disease in which arrhythmia occurs than when this condition is absent."

It would appear that the examiner should exercise great care to exclude any nervous excitement, make a thorough examination and report just what he finds, giving his opinions as to the cause of abnormalities and the character of the risk. Then let the home office make their decision. The following extract from a recent edition of "Suggestions to the Medical Examiners," of an old and prominent company, are well worth reading by every examiner: "The medical examiner should have constantly in mind two important differences in mental attitude between the patient and the applicant for insurance. In the first place many, especially at their first examination, or among the younger applicants, are extremely nervous; the idea that the examination may reveal some hidden ailment so takes possession of their minds as to disturb considerably the nervous equilibrium—to induce, as it were, a mild degree of shock. In such cases the pulse may be found extremely rapid or intermittent; there is apt to be pallor or muscular tremor. The picture is one of nervous debility or a want of normal bodily vigor. Whenever an examiner meets with this condition he should be able, by tactful handling, to reassure the applicant, and to re-establish the normal nervous balance; at any rate, he should make due allowance in his report for the disturbed mental state of his subject.

"As to the other difference to which we have referred, when he consults his physician the patient endeavors to describe every detail of the disease of which he complains. He conceals nothing. His mental attitude is one of unreserved co-operation. When he is a candidate for life insurance, the case is very different; his memory for details is less acute; his state of mind is one of antagonism. He believes himself to be a good risk, and his bias of mind in that direction is so strong as—no doubt unconsciously—to color his entire history. On this account a medical history for life insurance is a very different matter from that which is obtained from a patient. The patient assists his physician—the applicant for insurance does not assist the examiner. It requires time for any physician to adjust himself to this difference in mental attitude. The skilled medical examiner has learned this lesson."—*Pa. Medical Journal.*

---

### THE DANGER FROM LEPROSY.

In the *New York Medical Record* of January 27th last, under the title "Leprosy in Hawaii," I tried to show that there was less danger from the disease in Hawaii than there is here from our local infecting points. This article has been extensively copied into medical, literary and daily publications, and in the last misquoted and misrepresented for political purposes. I said:

"To a normal person of intelligence and moral and social discrimination, I should consider, so far as the danger of acquiring leprosy is concerned, that Hawaii, or any other country practicing segregation, offers substantial advantages."

An article copied into the Los Angeles *Times*, and credited to the Pittsburg *Dispatch*, says:

"According to Dr. Goodhue there are at present 1,100 cases of leprosy in Hawaii. The existence of this and other similar diseases in these and other newly acquired possessions or *protégés* of the United States will no doubt prevent many Americans from locating in these countries, but it does not prevent leprosy and other diseases from coming to this country."

The danger here is not because we have annexed Puerto Rico, Hawaii and the Philippines, but because the United States government, as well as the various State governments, has no specific laws providing either for the segregation of lepers in our midst, or against the spread of leprosy on the mainland. The trouble is with our attitude towards leprosy-infected countries and localities. We have been as indifferent to the diffusion of the disease here as we are in regard to the spread of the tubercular germ or of syphilis.

The danger of leprosy from Hawaii and Cebu, even now with our increasing trade relations and facilities for travel, is not so great as that from our contiguous neighbors, Mexico and Central America, where there are lepers; from the local *foci* in San Francisco, Louisiana, Minnesota, New Brunswick, and from individual cases in almost every State in the Union.

From these sources we have been for years, and are now, exposed to leprosy. In the *Medical Review* of a few weeks ago, Dr. McDougal of Ohio reports two cases in his practice in a little country town; and in the Philadelphia *Medical Journal* of March 16th, I see that a leper came to the Quaker City from Canada, having landed in that country from Barbadoes. A case was reported in this city last week. To one acquainted with the history of the disease in ancient times, the fact that we have scattered, and unknown, cases all over the country is startling; for leprosy was as prevalent at one time in Europe as measles is in this country to-day.

It was only by specific measures taken against it that it came to be stamped out, and we shall have it here epidemically perhaps, if we do not pass laws against its endemic existence.

This is not sentiment; it is truth. While there is no need for sensational newspaper articles, or hysterical action of any kind; while the annexation of new territory in the tropics has not increased the danger much, it has emphasized the need for prompt, comprehensive action on the part of our national government. We have allowed and are allowing lepers to pass freely over the country; to enter our borders from Mexico, Central America, the South American republics, and from the West Indes; to crowd to our shores from Norway, France and Italy. While we have general quarantine regulations, they have not applied to leprosy in any efficient way. The Scandinavian lepers went to Minnesota and other points of the west to give us lazarettos.

To be afflicted with leprosy is not a crime, but it is criminal for us to allow persons afflicted with the disease to expose others to it. When it comes to such quickly spreading diseases as smallpox, measles or scarlet fever, we isolate and punish infringement of our special laws. Leprosy, syphilis and tuberculosis are surely passed from one person to another; they are terrible diseases; and two of them are practically incurable. Yet because they are slow to show themselves; because cause and effect lie far apart, and defy correlation by easy methods; because the diseases are indolent in their manifestations, we remain lethargic, too, and do nothing to prevent their spread.

I would recommend, as I have before, that a commission be appointed to inquire into leprosy in this country; that this commission be empowered to visit other leprosy infected countries for the purpose of studying their methods of dealing with the disease; that the report and recommendations of this commission be acted upon without delay.

I see that Dr. Morrow, in his new work on leprosy has expressed the opinion that leprosy may become general in this country; this is in accordance with the history of the disease in other countries.

It thrives best in tropical countries perhaps, but it does well in very cold countries, as seen by its persistence in Norway, and in Minnesota and New Brunswick.

However, if the fancied danger of leprosy from Hawaii is going to rouse us to the real danger of leprosy in our midst, it will be another of the many adventages of annexing the islands to this country.—*By E. S. Goodhue, M.D., Los Angeles, Cal., in Medical Review.*

---

### VACANCIES TO BE FILLED IN U. S. MARINE HOSPITAL SERVICE.

A board of officers will be convened at the Service Building, 378 Washington street, New York city, Wednesday, May 23, 1900, for the purpose of examining candidates for admission to the grade of Assistant Surgeon in the United States Marine Hospital Service. Candidates must be between twenty-one and thirty years of age, graduates of a reputable medical college, and must furnish testimonials from responsible persons as to character.

The following is the usual order of the examination: 1. Physical. 2. Written. 3. Oral. 4. Clinical.

Candidates are required to certify that they believe themselves free from any ailment which would disqualify for service in any climate.

The examinations are *chiefly in writing*, and begin with a short autobiography of the candidate. The remainder of the written exercise consists in examination on the various branches of medicine, surgery and hygiene. *The oral examination* includes subjects of preliminary education, history, literature and natural sciences. *The clinical examination* is conducted at a hospital, and when practicable candidates are required to perform surgical operations on a cadaver.

Successful candidates will be numbered according to their attainments on examination, and will be commissioned in the same order as vacancies occur. Upon appointment the young officers are, as a rule, first assigned to duty at one of the large marine hospitals, as at Boston, New York, New Orleans, Chicago or San Francisco.

After five years' service, *Assistant Surgeons* are entitled to examination for promotion to the grade of *Passed Assistant Surgeon. Promotion to the grade of Surgeon* is made according to seniority, and after due examination as vacancies occur in that grade. Assistant Surgeons receive $1,600; Passed Assistant Surgeons receive $2,000, and Surgeons $2,500 a year. When quarters are not provided, commutation at the rate of thirty, forty or fifty dollars a month, according to grade, is allowed.

All grades above that of Assistant Surgeon receive longevity pay, ten per centum in addition to the regular salary for every five years' service up to forty per centum after twenty years' service.

The tenure of office is permanent. Officers traveling under orders are allowed actual expenses.

For further information, or for invitation to appear before the Board of Examiners, address Supervising Surgeon-General, U. S. Marine Hospital Service, Washington, D. C.

---

### TREATMENT OF NON-MALIGNANT GASTRIC AND DUODENAL ULCERS.

In so far as treatment is concerned, two things should be prominent in one's mind: first, to relieve immediate symptoms; second, to cure the ulcer. In hematemesis one cannot insist too strongly on rest in bed, and that the patient must not get up for any reason whatever. No food should be taken by the mouth, and in fact no liquid should enter the œsophagus. The lips may be bathed in water. If hemorrhage continues, some preparation of ergot should be used, perhaps followed by morphine in gr. $\frac{1}{4}$ doses. An ice- or cold-water bag should be applied to the stomach. In case of collapse, transfusion should be made with decinormal salt solution. During this period the patient should be fed by the bowel. Six ounces of peptonized milk should be given every three or four hours. At the end of three days a little liquid may be given by the mouth, *i. e.*, milk, lime water, beef tea, or the peptonoid. At the end of the week the patient should be put on a regular diet, and kept in bed. The bowels should be moved by laxatives, such as Apenta water. Warm applications should be made continuously to the epigastrium. After two weeks the patient may be allowed to get up, but food likely to distend the stomach should be avoided. The stomach should never be washed out or the tube used in gastric ulcer; profuse hemorrhage may occur with all its attendant dangers. There is, I am sure, much harm done sometimes by unskilful washing out of the stomach. Large pieces of mucous membrane may be caught in the eye of the tube and torn out. Bits of mucous membrane are so frequently found in the wash-water as to lead to the supposition that they were torn off by the tube. In fact, at post-mortem examination I have seen in a single case numerous erosions of the stomach caused by lavage. Operative measures have been frequently resorted to, and especially during the last few years.—THOMAS E. SATTERWHITE, M.D., New York, Consulting Physician to the Post-Graduate, Orthopedic, and Babies' Hospitals, in *Medical Record*.

